# Self-compassion moderates the relationship between neuroticism and depression in junior high school students

**DOI:** 10.3389/fpsyg.2024.1327789

**Published:** 2024-02-13

**Authors:** Ting Wang, Xicong Wu

**Affiliations:** ^1^Laboratory of Emotion and Mental Health, Chongqing University of Arts and Sciences, Chongqing, China; ^2^Chongqing No.110 Middle School, Chongqing, China

**Keywords:** depression, neuroticism, self-compassion, junior high school student, moderate

## Abstract

Neuroticism, an emotion-related personality trait, is often associated with a greater susceptibility to depression. On the other hand, self-compassion involves treating oneself in a peaceful, mindful, and friendly manner, particularly in the face of failure or frustration. The study investigated the relationship between neuroticism and depression in junior high school students, as well as the moderating role of self-compassion. A total of 757 junior high school students participated in the survey, which included the Children’s Depression Inventory, the Eysenck Personality Questionnaire-Revised, Short Scale for Chinese, and the Chinese version of the Self-Compassion Scale. The results revealed that neuroticism positively predicted depression, while self-compassion had a significant moderating effect on the relationship between neuroticism and depression. Specifically, higher levels of self-compassion were associated with a weaker link between neuroticism and depression. These findings suggest that interventions promoting self-compassion may be beneficial for students exhibiting neurotic tendencies.

## 1 Introduction

Junior high school students are in early adolescence. Due to the rapid physiological and psychological changes, they often have various mental health problems, particularly depression (Vander Stoep et al., 2011), which has been widely concerned by researchers. Depression will bring various adverse effects to the physical and mental development of junior high school students, including poor academic performance (Sörberg Wallin et al., 2019), more addictive behaviors (Gao et al., 2017), and even suicidal behavior (Hawton et al., 2013). At the same time, depression in junior high school may also increase the risk of unemployment, suicide, depression, and crime in adulthood (Vander Stoep et al., 2011). Neuroticism is a personality characteristic and a risk factor for depression in adolescence (Kotov et al., 2010). Self-compassion refers to an individual who treats himself in a peaceful, mindful and friendly way in the face of failure or frustration (Neff, 2003a). Previous studies showed that self-compassion was an effective protective factor for depression (MacBeth and Gumley, 2012; Krieger et al., 2013). However, there is currently no research on whether self-compassion moderates the relationship between neuroticism and depression. Therefore, the present study examined the relationship between neuroticism and depression in junior high school students and the moderating role of self-compassion.

In the five-factor model of personality, neuroticism represents a personality trait, which is related to emotional stability (Costa and McCrae, 1995). Highly neurotic people have unstable emotions (Yang et al., 2020), pay attention to negative emotions (Amin et al., 2004), and their self-schema is more negative (Otani et al., 2020). They tend to report more negative life events and cannot cope effectively, so they are more prone to emotional disorders and substance addiction. A longitudinal study on adolescents found that (Kercher et al., 2009), the initial neuroticism level will increase the probability of individuals suffering from negative life events, but also increase individuals’ negative cognition and thinking, and eventually lead to more depressive symptoms.

According to the cognitive vulnerability-transactional stress model (Hankin and Abramson, 2001), highly neurotic individuals are more likely to interpret neutral events in their lives as negative because they are more sensitive, and the way they deal with negative events triggers more negative events, making them more prone to depression (Rukh et al., 2023). Specifically, cognitively, highly neurotic individuals are easily attracted by negative emotional information and show negative processing bias (Amin et al., 2004), which is related to hypoesthesia, self-blame and more serious depression (Lam et al., 2003). In terms of emotional regulation, highly neurotic individuals are unable to carry out effective emotional regulation (Yang et al., 2020). When encountering negative events, they use more adverse emotional regulation strategies, such as rumination and expression inhibition (Lakdawalla and Hankin, 2008), which in turn aggravates their negative emotional experience and produces more depressive symptoms. Studies have indicated that neuroticism is a powerful predictor of depression, anxiety, and other emotional disorders (Chen et al., 2020; Barlow et al., 2021). Therefore, it is very important to find a way to break this vicious circle.

Self-compassion is a relatively new self-concept and intervention method, which consists of three basic components on bipolar: (1) self-kindness vs. self-judgment, which refers to being kind and understanding one’s own deficiencies and shortcomings, rather than criticizing severely; (2) Common humanity vs. isolation, refers to the realization that all people can make mistakes, and emphasizes the connection between people, rather than viewing one’s own mistakes as isolated experiences and feeling isolated from others; and (3) Mindfulness vs. over-identification, which refers to the awareness of the current painful feelings in a clear and balanced way, rather than becoming overly absorbed in them (Neff, 2003a). Previous studies have stated that self-compassion can protect individuals from frustrations, failures, and other painful negative events (Neff et al., 2007; Neff, 2011; MacBeth and Gumley, 2012), which provides psychological flexibility for coping with difficulties and adversity.

According to Neff (2003a), individuals with high self-compassion have more accurate self-cognition, including their own advantages and disadvantages, but they can recognize that defect is a part of human common experience, so they are more friendly and caring for themselves, rather than criticism and blame (Neff, 2011). Emotion regulation may be the psychological mechanism by which self-compassion works (Bakker et al., 2019). Individuals with high self-compassion will use more emotional acceptance (Zhang and Chen, 2016) and cognitive reappraisal strategies (Karl et al., 2018) when encountering failures or setbacks, and less rumination (Johnson and O’Brien, 2013) and avoidance tendencies (Krieger et al., 2013; Bakker et al., 2019). At the cognitive level, self-compassion priming can effectively reduce the subjects’ negative attentional bias (Yip and Tong, 2020). Thus, studies have found inverse relationships between self-compassion and depression in adult (MacBeth and Gumley, 2012), adolescent (Marsh et al., 2018), and chronic physical illness populations (Hughes et al., 2021). In longitudinal studies, self-compassion levels were found to predict depression 6 months later (Stutts et al., 2018; Neff, 2023), and even over a 5-year time span (Lee et al., 2021). Therefore, self-compassion is a protective factor for depression.

Previous studies have found a strong correlation between self-compassion and neuroticism (Neff et al., 2007; López et al., 2015). Some researchers have suggested that they may reflect the same underlying construct, albeit labeled differently (Pfattheicher et al., 2017). However, it is important to note that a strong correlation between constructs does not necessarily mean that they measure exactly the same thing (Neff et al., 2018). In fact, self-compassion has been shown to provide incremental validity relative to neuroticism in predicting life satisfaction and difficulties in emotion regulation (Neff et al., 2018). Additionally, neuroticism and self-compassion are conceptually different. Neuroticism emphasizes emotional instability and the resulting negative experiences, while self-compassion promotes a peaceful, non-judgmental, and self-accepting approach to setbacks, fostering self-forgiveness.

**Figure 1:**
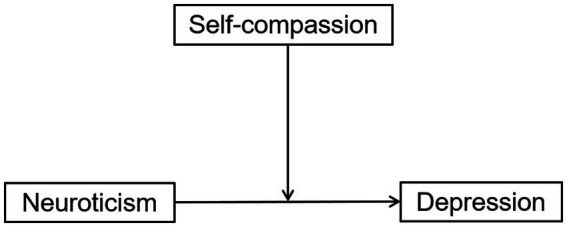
Relationship among the variables.

Given these differences, we believe that self-compassion and neuroticism are not the same constructs, and that self-compassion can serve as a protective factor for neuroticism. Because neurotic individuals are more sensitive, they tend to interpret neutral events as negative, and their way of handling things can exacerbate the situation, leading to a perception and experience of more negative events, resulting in depressive symptoms (Rukh et al., 2023). When a neurotic individual is able to handle perceived negative events in a self-compassionate manner, it can break the vicious cycle of neuroticism leading to depression. For example, Stutts et al. (2018) found that Self-compassion continues to alleviate the impact of perceived stress on depression even after 6 months. Therefore, we hypothesized that self-compassion moderates the relationship between neuroticism and depression (the hypothesized model is shown in Figure 1); the predictive effect of neuroticism on depression decreases when self-compassion levels are high.

## 2 Materials and methods

### 2.1 Participants and procedure

We recruited 806 participants from middle schools in southwest China. There were 16 classes in total, including seven classes in grade 7, six classes in grade 8, and three classes in grade 9. The survey was conducted in the classroom by the class’s mental health teachers, who were trained in advance. In class, the purpose and precautions of the research were explained to the students first, and then paper questionnaires were handed out to the students. After the students completed the questionnaires, the teachers collected them and handed them to the researchers after class. EpiData3.1 was used to input the questionnaire data by psychology students. 49 subjects were deleted due to too many missing values, and 757 valid data were finally obtained. Among them, there were 407 boys and 350 girls. Participants were aged between 11 and 16 years (mean age 13.42 ± 0.92). The study was approved by the Ethics Committee at Chongqing University of Arts and Sciences. We had obtained appropriate ethics committee approval for the research reported, and all subjects and their legal guardian gave written informed consent in our study. In addition, all methods were carried out in accordance with relevant guidelines and regulations.

### 2.2 Measures

#### 2.2.1 Children’s depression inventory

The scale has 27 questions to evaluate the situation of children in the past 2 weeks (Wu et al., 2010). Each item includes three sentences describing the degree of depression (such as “many things can bring me happiness,” “some things can bring me happiness,” and “nothing can bring me happiness”), which score 1, 2, and 3 points, respectively. The higher the score, the more serious the depression is. The CDI exhibits strong psychometric properties, including internal consistency, construct validity, and test re-test reliability across Chinese samples (Wu et al., 2010; Lei et al., 2022). The structure of the scale was verified using confirmatory factor analysis of the structural equation model, and model was estimated with the weighted least squares mean-adjusted and variance-adjusted estimator. The result indicated an acceptable model fit: *χ^2^/df* = 1.861, *p* < 0.001, Tucker Lewis index (TLI) = 0.960, comparative fit index (CFI) = 0.964, root mean square error of approximation (RMSEA) = 0.034. The Cronbach’s α coefficient of the scale in this study was 0.87.

#### 2.2.2 Eysenck personality questionnaire-revised, short scale for Chinese

The neuroticism subscale in eysenck personality questionnaire-revised, short scale for Chinese (EPQ-RSC) was selected to measure the neuroticism personality characteristics of junior high school students (Qian et al., 2000). The questionnaire including 12 items, involving various characteristics of neuroticism personality (such as “whether your emotions rise and fall from time to time”), and require the subjects to judge whether the sentence described them. The higher the score, the higher the neuroticism level is. The EPQ-RSC exhibits strong psychometric properties, including internal consistency, construct validity, and test re-test reliability across Chinese samples (Qian et al., 2000). The structure of the scale was verified using confirmatory factor analysis of the structural equation model, and model was estimated with the weighted least squares mean-adjusted and variance-adjusted estimator. The result indicated an acceptable model fit: *χ^2^/df* = 1.716, *p* < 0.001, Tucker Lewis index (TLI) = 0.989, comparative fit index (CFI) = 0.991, root mean square error of approximation (RMSEA) = 0.031. The Cronbach’s α coefficient of this subscale in this study was 0.84.

#### 2.2.3 Chinese version of self-compassion scale

The scale includes a total of 26 items and has six dimensions, i.e., self-kindness (five items, such as “When I’m feeling low, I make an effort to be more compassionate toward myself”), self-judgment (five items, such as “I have a critical and dissatisfied attitude toward my own shortcomings and inadequacies”), common humanity (four items, such as “When I encounter difficulties, I see them as a part of life, something that everyone goes through”), isolation (four items, such as “When I think about my own shortcomings, I tend to feel increasingly isolated and disconnected from the world”), mindfulness (four items, such as “When something painful happens, I try to look at the problem objectively”), and over-identification (four items, such as “When I’m feeling low, I tend to get caught up in things that are not going well”) (Chen et al., 2011). The questionnaire is scored on a five-point Likert scale, where 1 represents “never,” 2 represents “occasional,” 3 represents “sometimes,” 4 represents “often,” and 5 represents “always.” Negatively worded items (items on the Self-Judgment, Isolation, and Overidentification subscales) are reversed before calculating the total self-compassion score. Therefore, the higher is the score, the higher is the level of self-compassion. The CSCS exhibits strong psychometric properties, including internal consistency, construct validity, and test re-test reliability across Chinese samples (Chen et al., 2011; Li et al., 2022). The structure of the scale was verified using confirmatory factor analysis of the structural equation model, and model was estimated with the weighted least squares mean-adjusted and variance-adjusted estimator. The result indicated an acceptable model fit: *χ^2^/df* = 3.702, *p* < 0.001, Tucker Lewis index (TLI) = 0.906, comparative fit index (CFI) = 0.917, root mean square error of approximation (RMSEA) = 0.060. The Cronbach’s α coefficient of the scale in this study was 0.85.

## 3 Results

### 3.1 Descriptive statistics and correlation analysis

Descriptive statistics and Pearson correlation analysis were performed on neuroticism, self-compassion and depression. The results are shown in Table 1. There is a significant positive correlation between neuroticism and depression, a significant negative correlation between self-compassion and depression, and a significant negative correlation between self-compassion and neuroticism.

**Table 1:** Correlation matrix of neuroticism, depression and self-compassion (*n* = 757).

*M* ± *SD*		Neuroticism	Depression	Self-compassion
Neuroticism	16.11 ± 3.29	1		
Depression	37.40 ± 6.67	0.65^**^	1	
Self-compassion	83.02 ± 14.90	−0.47^**^	−0.48^**^	1

^**^p < 0.01.M, Mean; SD, Standard deviation.

### 3.2 Regressions

Using PROCESS Macro (Hayes, 2012), we entered neuroticism as the independent variable, self-compassion as the moderating variable, and depression as the dependent variable into Model 1. The results showed that (Table 2): Neuroticism positively predicts depression, and the interaction term between neuroticism and self-compassion is significant, indicating that the influence of neuroticism on depression is moderated by self-compassion.

**Table 2:** Results of the moderating effect.

Independent variable	*Coeff*	*se*	*t*	*F*	*R^2^*
Constant	−0.356	0.192	−1.860	228.474^***^	0.477
Neuroticism	1.028	0.062	16.484^***^		
Self-compassion	−0.100	0.013	−7.490^**^		
Neuroticism*self-compassion	−0.015	0.003	−4.599^***^		

^**^p < 0.01; ^***^p < 0.001.

**Figure 2:**
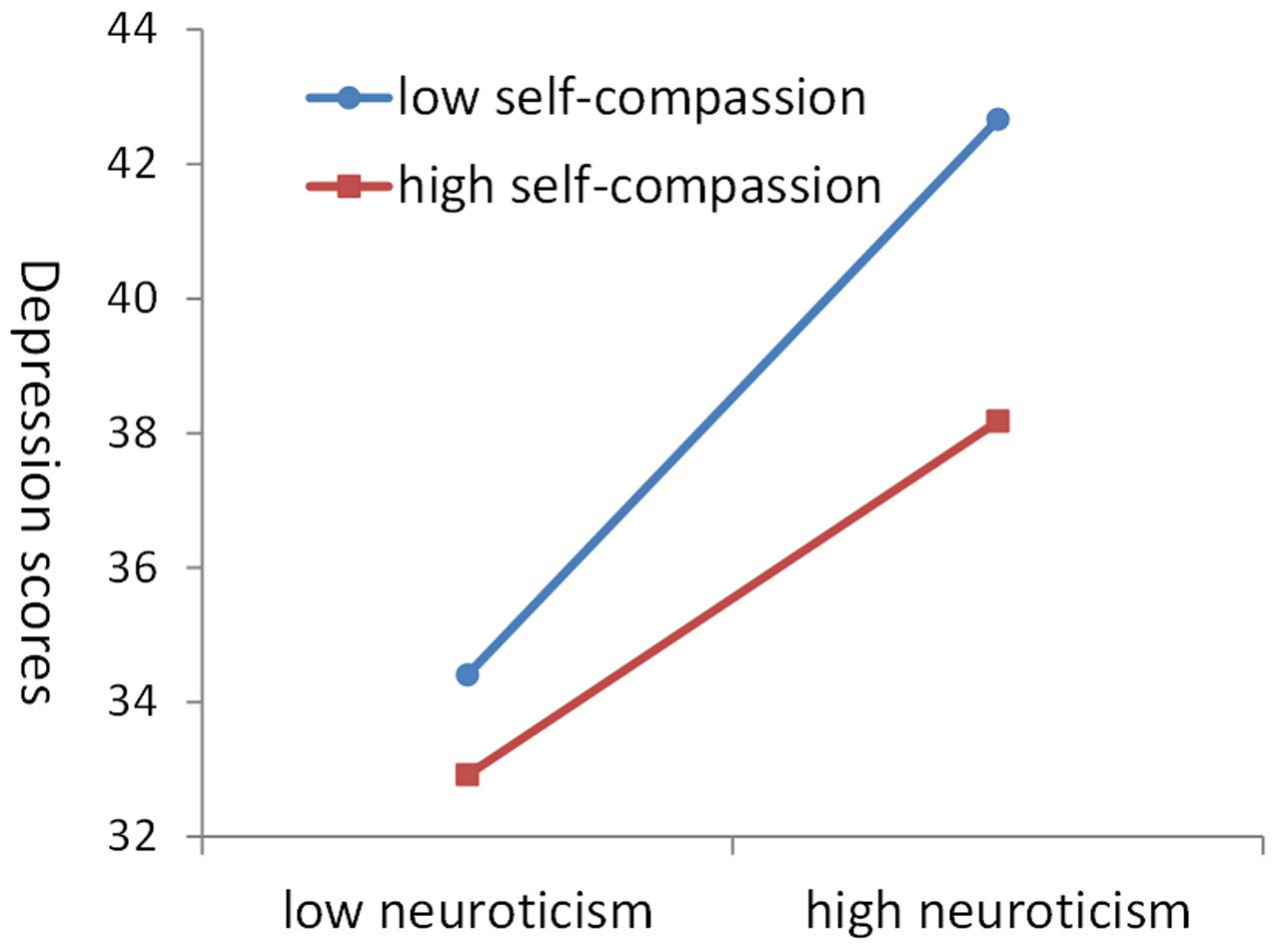
Interaction between neuroticism and self-compassion in predicting variance in depression.

In order to reveal the moderating effect more clearly, we conducted a simple slope test. The results showed that neuroticism positively predicted depression in students with low self-compassion (*β_simple_* = 1.26, *t* = 18.05, *p* < 0.001) and in students with high self-compassion (*β_simple_* = 0.80, *t* = 9.03, *p* < 0.001); more importantly, neuroticism was a weaker predictor of depression in students with high self-compassion, i.e., self-compassion played a protective role. Figure 1 depicted the regression line between neuroticism and depression when the self-compassion level was high and low (+1sd, −1sd) scores. We could see the regulatory effect of self-compassion on the relationship between neuroticism and depression: the higher the score of self-compassion, the lower the slope of the regression line, indicating that with the improvement of the level of self-compassion, the relationship between neuroticism and depression becomes weaker (Figure 2).

## 4 Discussion

In this study, 757 junior high school students were tested to investigate the predictive effect of neuroticism on depression, and the moderating effect of self-compassion on the relationship between them. The results showed that neuroticism can positively predict the depression of junior school students. The higher the neuroticism level, the higher the depression level of junior high school students; Self compassion played a moderating role between the two. For students with a high level of self-compassion, neuroticism has a weak predictive power for depression. This showed that self-compassion played a protective role in the influence of neuroticism on depression, and it may be possible to prevent depression by improving the level of self-compassion of students with neurotic trait.

Neuroticism could positively predict depression in junior high school students. According to the cognitive vulnerability-transactional stress model (Hankin and Abramson, 2001), individuals with high neuroticism are more likely to cause negative reactions of their peers in some way, so as to experience more negative life events. At the same time, they will respond to these events with negative emotions and negative thinking, leading to the increase of depressive symptoms (Kercher et al., 2009). For example, a junior high school student with high neuroticism was more likely to overreact to a joke, resulting in negative feedback from his peers. At this time, negative life events increased and negative emotions increased, which lead to more rumination. This made individuals difficult to effectively regulate negative emotions, leading to more depressive symptoms. Recent neuroimaging studies (Yang et al., 2020) have found that neuroticism levels are significantly negatively correlated with the activity of the dorsomedial prefrontal cortex (dmPFC), the ventromedial prefrontal cortex, and the medial prefrontal cortex, which regulate negative emotions. It is also negatively correlated with the connectivity between the amygdala and dmPFC, which regulates negative emotions.

The present study found that self-compassion played a moderating role between neuroticism and depression, and the predictive effect of neuroticism on depression is weakened in junior high school students with high level of self-compassion. This showed that self-compassion may help to reduce the depressive symptoms of junior high school students who showed a high level of neuroticism. The role of self-compassion may be reflected in cognitive processing and emotional regulation. From the perspective of cognitive processing, self-compassion priming has been shown to reduce the negative attention bias in individuals (Yip and Tong, 2020), thereby helping neurotic individuals to adopt a less negative perspective when encountering adverse events in life. In terms of emotional regulation, individuals with a high level of self-compassion showed less rumination, less emotional avoidance, and applied more effective emotional regulation strategies (Bakker et al., 2019). That is, self-compassion could help individuals use appropriate emotion regulation strategies to deal with frustration events, so as to break the cycle from neuroticism to depression and alleviate depressive symptoms.

Some researchers have suggested that self-compassion and neuroticism may reflect the same underlying construct (Pfattheicher et al., 2017). However, the study found a moderate correlation (*r* = −0.47) between self-compassion and neuroticism, and both self-compassion and the interaction between self-compassion and neuroticism contributed to the prediction of depression. Therefore, it demonstrates that they are two independent constructs. From the perspective of core and surface characteristics (Kandler et al., 2014), self-compassion and neuroticism are different at the hierarchical level. Neuroticism is considered a core feature, a pattern of thoughts, feelings, and behaviors that is not easily changed over time or situations. In contrast, self-compassion is considered a surface feature, appearing later and being less stable than core features, and can be cultivated and strengthened through various interventions such as training programs (Ferrari et al., 2019; Croft and Byrd, 2023; Neff, 2023). Empirical research evidence seems to be consistent with this view. Neuroticism is more associated with genetics, while self-compassion is more influenced by the environment, such as attachment style (Arambasic et al., 2019), childhood abuse (Miron et al., 2016), and sociocultural differences (Neff, 2023). Therefore, self-compassion appears to be a less stable, more environmentally influenced surface manifestation of personality.

From the perspective of traits, self-compassion is an attitude to deal with setbacks and misfortunes in a peaceful, mindful and friendly way (Neff, 2003b). From the perspective of intervention, self-compassion is an emotional and cognitive adjustment strategy (Germer and Neff, 2013), including treating yourself with mindfulness and friendliness and recognizing the common characteristics of human nature. From the perspective of trait, this study found that self-compassion plays a protective role in the prediction of neuroticism on depression, so self-compassion intervention for neurotic junior school students to improve their level of self-compassion could protect them from depression. Therefore, future research can further investigate the effect of self-compassion intervention on highly neurotic individuals.

This study has some limitations. First, all participants in this study were recruited from China. Whether the result reported herein is influenced by different cultural backgrounds needs to be further investigated. Second, this study is only a cross-sectional study, and further experimental or interventional studies are needed to test its conclusions. In the future, self-compassion interventions for neurotic individuals and the long-term effects should be further investigated. Finally, we did not explore what factors affect a student’s level of self-compassion. Future study could focus on this topic in the context of Chinese culture.

Despite some shortcomings, the present study still has potential clinical and practical implications. Studies have demonstrated that interventions based on self-compassion can relieve depression and anxiety (Galla, 2016). Our findings broaden the implications of self-compassion interventions, suggesting that they should be applied to specific at-risk students, for example, by providing coping strategies or emotional regulation for students with highly neurotic.

## 5 Conclusion

The present study showed that the higher the neuroticism level, the more likely the junior high school students are to have depressive symptoms; the higher the level of self-compassion, the smaller the predictive effect of neuroticism on depression. Therefore, self-compassion interventions should be applied to junior high school students with highly neurotic.

## Data availability statement

The raw data supporting the conclusions of this article will be made available by the authors, without undue reservation.

## Ethics statement

The studies involving humans were approved by Ethics Committee of Chongqing University of Arts and Sciences. The studies were conducted in accordance with the local legislation and institutional requirements. Written informed consent for participation in this study was provided by the participants’ legal guardians/next of kin.

## Author contributions

TW: Data curation, Investigation, Methodology, Writing – original draft, Writing – review & editing. XW: Data curation, Validation, Writing – original draft.
